# Wharton’s jelly mesenchymal stem cells attenuate global hypoxia-induced learning and memory impairment via preventing blood-brain barrier breakdown

**DOI:** 10.22038/IJBMS.2023.70137.15250

**Published:** 2023

**Authors:** Tahmineh Mokhtari, Maryam Shayan, Amirmohammad Rezaei Rashnudi, Gholamreza Hassanzadeh, Kobra Mehran Nia

**Affiliations:** 1 Hubei Key Laboratory of Embryonic Stem Cell Research, Faculty of Basic Medical Sciences, Hubei University of Medicine, Shiyan, Hubei, People’s Republic of China; 2 Department of Histology and Embryology, Faculty of Basic Medical Sciences, Hubei University of Medicine, Shiyan, Hubei, People’s Republic of China; 3 School of Medicine, Tehran University of Medical Sciences, Tehran, Iran; 4 Department of Anatomy, School of Medicine, Tehran University of Medical Sciences, Tehran, Iran; 5 Department of Neuroscience and Addiction Studies, School of Advanced Technologies in Medicine, Tehran University of Medical Sciences, Tehran, Iran

**Keywords:** Blood-brain barrier, Hippocampus, Hypoxia, Intraventricular, Mesenchymal stem cells, Wharton jelly

## Abstract

**Objective(s):**

Intracerebroventricular (ICV) injections of mesenchymal stem cells (MSCs) may improve the function and structure of blood-brain barrier (BBB), possibly by preserving the BBB integrity. This study examined the impact of Wharton’s jelly (WJ)-MSCs on cognitive dysfunction and BBB disruption following a protracted hypoxic state.

**Materials and Methods:**

Twenty-four male Wistar rats were randomly studied in four groups: Control (Co): Healthy animals, Sham (Sh): Rats were placed in the cage without hypoxia induction and with ICV injection of vehicle, Hypoxic (Hx)+vehicle: Hypoxic rats with ICV injection of vehicle (5 μl of PBS), and Hx+MSCs: Hypoxic rats with ICV injection of MSCs. Spatial learning and memory were evaluated one week after WJ-MSCs injection, and then animals were sacrificed for molecular research.

**Results:**

Hypoxia increased latency and lowered the time and distance required reaching the target quarter, according to the findings. Furthermore, hypoxic rats had lower gene expression and protein levels of hippocampus vascular endothelial (VE)-cadherin, claudin 5, and tricellulin gene expression than Co and Sh animals (*P<*0.05). Finally, administering WJ-MSCs after long-term hypoxia effectively reversed the cognitive deficits and prevented the BBB breakdown via the upregulation of VE-cadherin, claudin 5, and tricellulin genes (*P<*0.05).

**Conclusion:**

These findings suggest that prolonged hypoxia induces spatial learning and memory dysfunction and increases BBB disruption, the potential mechanism of which might be via reducing VE-cadherin, claudin 5, and tricellulin genes. Hence, appropriate treatment with WJ-MSCs could reverse ischemia adverse effects and protect the BBB integrity following prolonged hypoxia.

## Introduction

The result of strong connections among the endothelial cells in the arteries of the central nervous system (CNS) is the blood-brain barrier (BBB) (1). To create a stable environment for the CNS, this barrier plays an essential role in ionic regulation controlling the entry of neurotransmitters, including glutamate (2, 3), prevents the access of plasma macromolecules such as albumin and prothrombin which induce apoptosis and prevents the entry of neurotoxins into the CNS (4, 5). BBB provides an optimal microenvironment for neural and synaptic activity (6). This highly developed barrier is characterized by two major junctional complexes, including tight junction (TJ) and adherens junction (AJ), between endothelial cells of the brain microvasculature. In the BBB, AJs are highly differentiated cells that lie beneath TJs, initiate cell-cell adhesion, and connect to the underlying actin cytoskeleton (7, 8). TJs are composed of claudin (CLDN, with 27 members) and TJ-associated MARVEL proteins (TAMP, e.g., occludin, tricellulin, and marvelD3) (9, 10). Tricellulin is a protein found in the tricellular junction which helps maintain the impermeability of the BBB to macromolecules (11). All these proteins strengthen the BBB, and disruption in any of them causes BBB malfunction that results in irreversible complications (12). 

Vascular endothelial (VE)-cadherin is one of the most important AJ proteins, establishing a firm bed for TJ formation (13). TJ proteins have been detected in the blood samples of patients with stroke and cerebral bleeding (14, 15). Hypoxia is one of the most common causes of BBB degradation, although its processes are poorly understood. Long-term exposure to hypoxia increases reactive oxygen species (ROS) levels and induces glutathione oxidation (16). ROS are important signaling molecules activated in response to oxidative stress (17). Increased levels of ROS lead to damage cellular structures like nucleic acids, proteins, and lipids (18). Decreasing the oxygen level in terms of hypoxia activates HIF-1a factor, and consecutively VEGF is activated. It causes tight junction dysfunction and increases the permeability of the BBB (19, 20). Hypoxia, on the other hand, is linked to enhanced macrophage production of pro-inflammatory molecules such as interleukin-1β (IL-1β) (21). This cytokine may cause BBB disruption and increased permeability by phosphorylating TJ proteins, producing barrier dysfunction (22). It can have a similar effect by altering the expression level of the CLDN5 gene. Besides, increasing MMPs, especially MMP-9, can cause loss of tight junction (23). The rupture of the BBB is linked to a number of pathological processes, including an influx of pathogens and neurotoxic chemicals into the brain, which causes neuroinflammation and neuronal death, ultimately leading to neurodegenerative disorders, such as Alzheimer’s disease (AD) (24). Recently, it was shown that prolonged hypoxia could increase the levels of proinflammatory cytokines (e.g., tumor necrosis factor alpha [TNF-α] and IL-1β) that induce AD-like symptoms in an animal model (25). 

Mesenchymal stem cells (MSCs) are tissue-specific progenitor cells with self-renewal abilities found in various tissues, including bone marrow, adipocytes, and fetal umbilical cord (26). Due to their immunosuppressive, anti-inflammatory, and powerful paracrine capabilities, MSCs are able to modulate immune responses through inflammation regulation and assist in injury healing rather than developing into neurons and glial cells (27, 28). Cell therapy using MSCs or their derivatives was confirmed to treat several neurological diseases, including spinal cord injury (29), AD (30), Parkinson’s disease (31), multiple sclerosis (32), and stroke (33). Umbilical cord (UC) has distinguished itself as a special source of MSCs from other sources by offering a number of benefits. It is noteworthy that MSCs can be found in both UC matrix, also known as Wharton’s jelly (WJ), and UC blood (UCB), but they are significantly more prevalent in WJ tissue (34, 35). For the first time, Thomas Wharton (1956) has defined WJ as a mucous connective tissue of the UC between umbilical vessels and the amniotic epithelium (36). WJ-MSCs were first isolated by McElreavey *et al.* in 1991 (37). According to the literature, WJ-MSCs can be used for the treatment of a variety of conditions, including neurological disorders, orthopaedic injuries, kidney injuries, liver injuries, lung injuries, and cancer (38). Increasing the concentration of therapeutics in the CNS can be accomplished by using intracerebroventricular (ICV) therapy (39). The advantageous ICV route may be due to achieving a widespread distribution of MSCs in the parenchyma of brain (40, 41). Furthermore, injection of stem cells into the lateral ventricles was reported as a safe, feasible and well-tolerated method in animal and human studies (42, 43). 

 Our study aimed to investigate the influence of ICV microinjection of Wharton’s jelly (WJ)-MSCs on cognitive deficits in terms of BBB breakdown induced by prolonged hypoxia. For this purpose, the following examinations were performed; 1) Spatial cognitive behavior in Morris water maze (MWM), 2) Molecular studies (gene expression and protein levels of tricellulin, VE-cadherin, and CLDN5).

## Materials and Methods

***Study design and animals***

**Figure 1:**
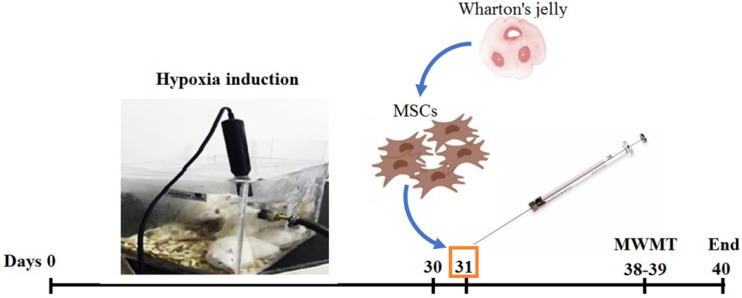
Experimental protocol design and timeline for assessing the effects of WJ-MSC on hypoxia-induced cognitive disruption

In the present study, 24 male Wistar rats (200-240 gr) were obtained from Pasteur Institute, Tehran, Iran. The animals were kept in the Anatomy Department of Tehran University of Medical Sciences in a standard condition (a 12 hr:12 hr light: dark cycle and a temperature of 23±2 ^°^C) with free access to water and food. All experimental protocols were carried out in accordance with the National Institutes of Health’s Global Animal Welfare Guidelines and the ethics committee at Tehran University of Medical Sciences in order to employ the fewest number of animals and cause the least amount of suffering (Ethical code: IR.TUMS.MEDICINE.RES.1395.300). After gaining approval from the kid’s relatives and confirming that the youngster was healthy, the umbilical cord was removed from the hospital. The animals (n=24) were randomly divided into four groups: 1) Control (Co): Healthy animals; 2) Sham (Sh): Rats were placed in the cage without inducing hypoxia and received ICV injection of vehicle (PBS, 5 μl); 3) Hypoxic (Hx)+vehicle: Hypoxic animals received ICV injection of vehicle; and 4) Hx+WJ-MSCs: Animals became hypoxic and received ICV injection of WJ-MSCs. Animals were examined Tested for spatial cognitive behavior in MWM one week after ICV injection of vehicle or stem cells. Finally, they were sacrificed under deep and irreversible anesthesia, and brain tissue samples were used for molecular evaluations (Figure 1). 

***Induction of hypoxia model***

As described in the previous study, the animals were exposed to 8% oxygen and 92% nitrogen for 4 hr daily in a Plexiglass hypoxia chamber (20×20×30 cm^3^), which was equipped with an oxygen/nitrogen pressure control device and measured by oxygen O2 m (lutron DO-5510 O2 m, Taipei, Taiwan). Animals were exposed to hypoxia by placing rats in the specific cage for 30 days (4 hr/day). Animals were kept in standard cages during non-hypoxic hours (25).

***Isolation, culture, and identification of WJ-MSCs***

Human UC samples were aseptically collected from full-term delivery by cesarean section at the Arash Women’s Hospital affiliated to Tehran University of Medical Sciences. Informed consent was received from mothers. Wharton’s jelly from the umbilical cord was used to separate MSCs. Briefly, Umbilical cords from full-term pregnancies were prepared. After the vascular structures were taken out, the UC was cut into 2 mm^2^ explants. Then, these specimens were cultured in Dulbecco’s modified Eagle’s medium (Gibco, USA) with F12 for one week. In addition, fetal bovine serum (15%, Gibco, USA), penicillin (100 U/Ml), amphotericin B (1 μg/ml, Gibco, USA), and streptomycin (100 μg/mg, Gibco, USA) were added. The medium was exposed to a humidified incubator (5% CO_2_ & 37 ^°^C). After observing outgrowth of cells, the residuals were removed, and the medium was changed every 3 days. To achieve a 90% confluence, this method was repeated numerous times. WJ-MSC was then isolated using trypsin ethylene diamine tetraacetate (0.25 percent, Gibco, USA). MSCs were collected and utilized for tests after the third or fourth passage. 

***WJ-MSCs transplantation and labeling***

In the experimental groups, under stereotaxic surgery, 1×10^5^ cells were injected into the both ventricles using 5 μl injection of PBS as a vehicle. Brains were dissected under full anesthesia one day following behavioral testing, and hippocampi tissues were extracted. The samples were deposited in 1.5 microtubes, quickly frozen in liquid nitrogen for 30 seconds, and maintained at -80 ^°^C until the proteins and genes were analyzed. The right hippocampal specimens were considered for Western blot analysis, and the left hippocampal samples for RNA extraction.

***Morris water maze test***

One week after ICV injection of stem cells, the spatial cognitive functions of rodents can be evaluated using Morris Water Maze (MWM) test (44). The maze consisted of the pool from a round black tank (Diameter:140 cm & Height: 60 cm) filled with nontoxic water (depth: 35 cm, temperature: 24±2 ^°^C). The pool was divided into the four equal areas and four prominent visual cues were put on each side of four quadrants. A hidden platform (10 cm in diameter) was located 2 cm below the water surface in the target quadrant. Each animal was assessed using a one-day training program. After 1.5 hr of full animal training, the behavior of each rat was videotaped and analyzed for parameters, including time spent in the target quarter, distance traveled in the target quadrant, escape latency, and total traveled distance. One day before the preliminary test, animals were habituated to the pool by allowing a 30 s free swim while the platform was removed (45). 

***Tissue preparation***

Under anesthesia, the entire fresh brain was removed from each rat and placed in ice-cold PBS (pH=7.4). The thalamic tissue of one hemisphere was peeled out to reveal the ventricular surface of hippocampal tissue after the brain was divided along the mid-sagittal plane. Along the length of hippocampal fissure, the entire hippocampus was removed using a spatula tip (46)*.*

***Gene expression***

The hippocampal tissue (n=3 in each group) were used to perform quantitative real-time PCR (qRT-PCR). Briefly, this technique was carried out to determine the gene expression of CLDN5, VE-cadherin, and tricellulin in each group. First, the primer was designed using Generunner software. Next, the total RNA was extracted from the hippocampi tissues using TriPure™ isolation reagent (Sigma-Aldrich) and converted to cDNA. The cDNA was subsequently amplified using real-time PCR machine and the expression of the specified genes was evaluated. Each reaction was performed according to the manufacturer’s instructions using SYBR Green Master Mix (Applied Biosystems, Foster City, CA), and the RNA of each sample was transcribed to cDNA using a reverse transcriptase kit (MWG-Biotech, Germany). GAPDH was utilized as a housekeeping gene, and the fold change was computed as relative gene expression via the 2-ΔΔCT method. The PCR primers are listed in Table 1.

***Western blotting***

Protein lysis buffer was used to lyse hippocampi tissues (Sigma, St. Louis, MO, USA). The, the samples were and centrifuged at 15,000 rpm. Protein concentration was measured by loading the samples onto polyacrylamide gels containing 8-15 percent sodium dodecyl sulfate. Afterward, the samples were moved to polyvinylidene difluoride (PVDF) transfer membranes (Sigma, USA) and incubated with primary antibodies against CLDN5 (1:1000, Abcam), VE-cadherin (1:1000, Abcam), and tricellulin (1:1000, Abcam) overnight at 4 ^°^C and 1 hr for secondary antibody detection (chemiluminescence reagent; Santa Cruz Biotechnology, Santa Cruz, USA).

***Statistical methods***

SPSS 19.0 (IBM SPSS Inc., New York, NY, USA) and GraphPad Prism 5.0d (GraphPad Software, La Jolla, CA, USA) were used for data analysis (GraphPad Software, La Jolla, CA, USA). To compare the means of the experimental groups, normal data were examined using one-way analysis of variance (ANOVA), t-test, and Tukey’s *post hoc* test; otherwise, the nonparametric Kruskal-Wallis test was used. A significance level of 0.05 was considered. Data are presented as mean±standard deviation (SD).

## Results

***Flow-cytometry results***

**Figure 2:**
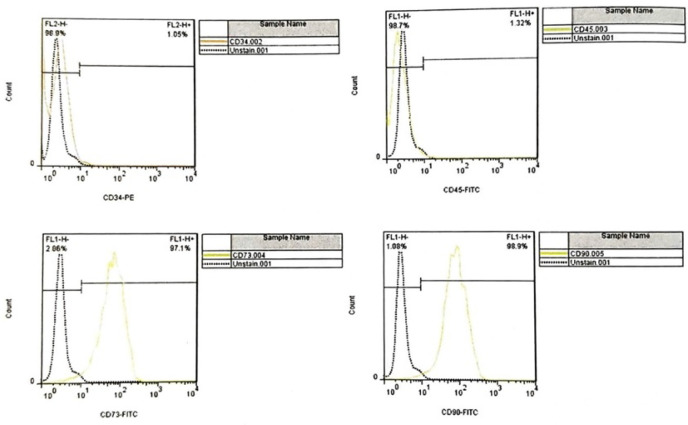
Flow-cytometry results to assess the CD surface markers of WJ-MSCs. A downward trend in CD34 and CD45 and an upward trend in CD 73 and CD90 by flow-cytometry assay

Flow cytometry results in WJ-MSCs were examined for surface markers of umbilical cord matrix mesenchymal cells after three passages to confirm mesenchymal markers. For this purpose, CD34 (1.05%) and CD45 (1.32%) were used as negative markers, and CD73 (1.97%) and CD90 (9.98%) were used as positive markers, which confirms mesenchymal cells (Figure 2).

***Effect of WJ-MSCs on behavioral alterations***

**Figure 3:**
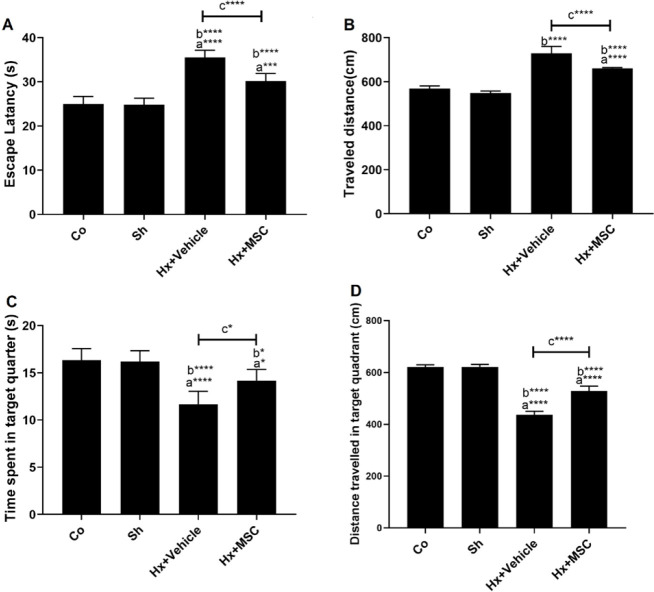
The effects of ICV injection of MSCs on behavioral alteration of hypoxic rats A) Escape latency, B) Time spent in target quarter, C) Total traveled distance, and D) Distance traveled in the target quadrant; a vs. Co group, b vs. Sh group, c vs. Hx+vehicle group. * *P<*0.05, ** *P<*0.01, *** *P<*0.001, **** *P<*0.0001

ICV: Intracerebroventricular; MSCs: Mesenchymal stem cells; CO: Control; Sh: Sham; Hx: Hypoxia

The MWM test was used to evaluate the spatial learning and memory capabilities of the animals. The escaping latency (F (3, 20)=57.86) and total traveled distance (f (4, 10)=41.1) of hypoxia-exposed rats were significantly greater than those of Co and Sh animals (*P*<0.05, Figure 3 A, B). Furthermore, decreased time spent (F (3, 20)=18.79) and distance traveled (F (3, 20)=275.6) in the target quarter were observed in hypoxic animals (*P<0.05*, Figure 3 C, D). The ICV administration of MSCs significantly alleviated the learning and memory dysfunctions compared with Hx+vehicle group (*P<0.05*, Figure 3 A-D). 

***Effect of WJ-MSCs on the hippocampal gene expression***

**Figure 4:**
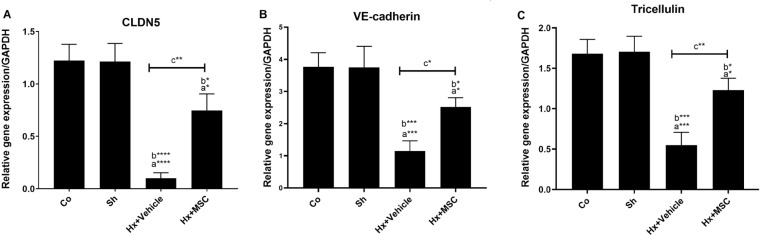
The effects of ICV microinjection of MSCs on the gene expression of hippocampal A) CLDN5, B) VE-cadherin, and C) Tricellulin hypoxic rats; a vs. Co group, b vs. Sh group, c vs. Hx+vehicle group. * *P<*0.05, ** *P<*0.01, *** *P<*0.001, **** *P<*0.0001

ICV: Intracerebroventricular; MSCs: Mesenchymal stem cells; CO: Control; Sh: Sham; Hx: Hypoxia

A significant decrease in the gene expression of CLDN5 (F (3, 8)=41.54,* P<0.05*, Figure 4A), VE-cadherin (F (3, 8)=23.08,* P<0.05*, Figure 4B), and Tricellulin (F (3, 8)=30.39,* P<0.05*, Figure 4C) was observed in Hx+vehicle and Hx+MSC groups compared to Co and Sh groups. However, MSCs could significantly increase the hippocampal CLDN5, VE-cadherin, and Tricellulin gene expression in the Hx+MSC group compared to the Hx group (*P<0.05*, Figure 4 A-C).

***Effect of WJ-MSCs on the hippocampal Protein Concentration***

**Figure 5:**
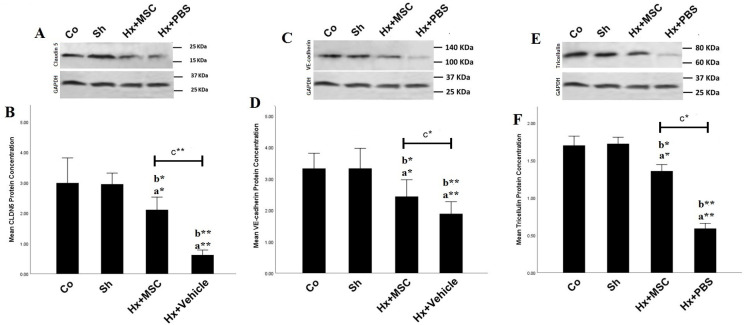
The effects of ICV microinjection of MSCs on the protein levels of hippocampal A) CLDN5, B) VE-cadherin, and C) Tricellulin hypoxic rats; a vs. Co group, b vs. Sh group, c vs. Hx+vehicle group.* *P<*0.05, ** *P<*0.01, *** *P<*0.001, **** *P<*0.0001

ICV: Intracerebroventricular; MSCs: Mesenchymal stem cells; CO: Control; Sh: Sham; Hx: Hypoxia

The hippocampal CLDN5 (F (3, 8)=65.91, *P<0.05*, Figure 5 A, B), VE-cadherin (F (3, 8)=30.33, Figure 5 C, D), and Tricellulin (F (3, 8)=207.7, *P<0.05*, Figure 5 E, F) protein levels were significantly different in study groups. A significant decrease was observed in CLDN5, VE-cadherin, and Tricellulin protein levels in the Hx+veh and Hx+MSC groups compared to the Co and Sh groups (*P<0.05*). However, ICV injection of MSCs could significantly improve the protein levels of hippocampal CLDN5, VE-cadherin, and Tricellulin in the Hx+MSC group compared to Hx+vehicle (*P<0.05*, Figure 5 A-F).

## Discussion

This research explored the effect of MSCs isolated from human WJ on spatial cognitive impairment and BBB disruption induced by chronic hypoxia. Our findings demonstrated that the spatial learning and memory of animals were compromised by global hypoxia. The results of the MWM test showed that exposure to hypoxia had an impact on learning and memory capacity by lengthening the latency time and distance traveled and shortening the time and distance spent in the target quarter. Emerging evidence has shown that hypoxia leads to memory deficits in the CNS. Anxiety, depression, anxiety, and neurodegeneration are all associated with this condition (47). There is a correlation between the severity of cognitive deficit and the length and level of hypoxia (48). Similar studies also have proved that exposure to ischemic condition can increase latency time and distance traveled ad reduce time and distance spent in the target quarter (44, 49). 

 VE-cadherin, CLDN5, and tricellulin gene expression and protein levels of hippocampal VE-cadherin, CLDN5, and tricellulin were decreased, confirming a link between behavioral impairment and BBB degradation. These proteins are predominant in the construction of TJs and AJs, which play crucial roles in the impermeability of BBB (50). Several pathological events were described for hypoxia-induced cognitive dysfunction. Recently, the results of an animal study showed that hypoxia induces AD-like dementia symptoms, e.g., memory dysfunction via the dysregulation of seladin-1 and Tuj1 in the hippocampal region correlated with enhanced serum levels of TNF-α and IL-1β (25). In another study, cognitive disability, neuroinflammation, and accumulation of amyloidβ/p-Tau were observed following repeated hypoxia exposure (51). On the other hand, hypoxia may disrupt the function of BBB proteins by increasing ROS levels and activating many factors, including cytokines (19). Inhibiting myosin light chain kinase has been proven to successfully minimize hypoxia-induced BBB disruption in previous investigations (52). Neuroinflammation in terms of increased levels of cytokines is one of the most critical consequences of hypoxia, which can lead to BBB dysfunction in various ways. During hypoxia, ROS-mediated activation of the hypoxia-inducible factor 1 (HIF-1) signaling pathway stimulates nuclear factor kappa B (NF-κB) in the microglial cells (53, 54). Activation of NF-κB pathways are associated with the upregulation of cytokines, causing more tissue damage (55). Increased cytokine levels may affect BBB protein activities, leading to lower trans-epithelial electrical resistance (TEER) and BBB collapse (56, 57). Moreover, it was shown that vascular leakage and brain edema were associated with disruption of continuity between occludin and zonula occludens-1 (Zo-1) under hypoxia. This event was medicated by dysregulation of matrix metalloproteinase 9 (MMP9) by a mechanism involving vascular endothelial growth factor (VEGF) (23). To show the essential role of TAMPs, hypoxia was inducted to redistribute tricellulin from three- to two-cell contacts, confirming that this protein is a regulator of junctional redox for hypoxia-associated changes (58). BBB disintegration was partially attributable to caveolin 1 (CAV1)-mediated redistribution of membranous CLDN5 into the cytosol during hypoxia, according to both *in vitro* and *in vivo* (zebrafish) investigations (59) .We used MSCs to alleviate hypoxia-induced brain dysfunction. Our results showed that ICV administration of MSCs could successfully improve learning and memory deficits in hypoxic rats. While growing evidence has indicated the transformation of MSCs naturally into other cells, e.g., osteocytes, chondrocytes, adipocytes, myocytes, astrocytes, neurons, and endothelial cells (60). Nonetheless, the paracrine functions of MSCs in the production of growth factor and immunosuppressive substances have drawn the attention of researchers (61). According to the findings of various research, WJ-MSCs induced neuronal survival by secreting neurotrophic substances, enhancing neurogenesis, and improving the axon survival ratio (62, 63). MSCs can significantly reduce inflammatory factors and increase anti-inflammatory cytokines (64). Moreover, MSCs were reported to improve neurological deficits associated with hypoxic conditions via a wide range of mechanisms. Following hypoxic ischemic encephalopathy, ICV injection of human umbilical cord-derived MSCs could exhibit neuroprotective effects via suppressing apoptosis and regulating the secretion of TNF-α and IL-1β (65). 

Our study confirmed that the gene expression and protein levels of VE-cadherin, CLDN5, and tricellulin were reduced in the hippocampus one week after the injection of MSCs. These findings showed that MSCs might help rats recover from BBB disruption caused by hypoxia. MSCs have the ability to stabilize endothelial barrier functions, although the processes by which they do so far unknown. Intracranial injection of MSCs was reported to prevent BBB leakage via regulation of intercellular adhesion molecule-1 (ICAM-1) and MMP-9 in a mice model of stroke (66). Moreover, umbilical cord-derived MSCs could alleviate BBB disrupted permeability via reversing the decreased levels of TJ proteins, including CLD5, occludin, and ZO-1 (67). Under the hypoxic condition, it was indicated that ICV injection of WJ-MSCs diminished the levels of inflammatory factors TNF-α, IL-1β, and IL-18 and regulated the gene expression and protein levels of VEGF, resulting in reduced neural death in the hippocampus (68). MSCs were also demonstrated to lower BBB permeability by increasing filament density in astrocyte legs, which reduced BBB permeability (69). Human MSCs were shown to reduce vascular permeability in an *in vitro* investigation via modulating the VE-cadherin and β-catenin pathways (70).

**Table 1:** Sequences of Primers in qRT-PCR

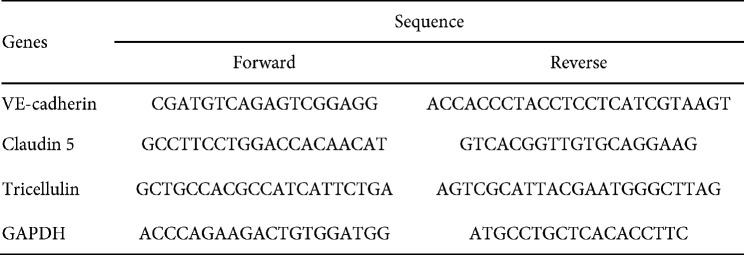

CD: Cluster of Differentiation, WJ-MSCs: Wharton’s jelly-Mesenchymal stem cells

## Conclusion

This study showed that hypoxia led to cognitive deficits that might be associated with the BBB disruption in terms of the disarrangement of TJ and AJ proteins (tricellulin, VE-cadherin, and CLDN5). ICV injection of WJ-MSCs reversed the adverse effects of hypoxia (learning and memory dysfunction) and protected the integrity of BBB. 

## Authors’ contributions

G H and K M contributed substantially to the conception and design of the study. G H, T M, M S, and A RR contributed to perform the experiment, T M contributed to analyze the data, T M, A RR, and KM drafted or provided critical revision of the article. G H provided the final approval of the version to publish. All authors discussed the results and contributed to the final manuscript.

## Conflicts of Interest

The authors declare that they have no competing interests.
